# Peripheral immune profiling in frontotemporal dementia

**DOI:** 10.1093/braincomms/fcag089

**Published:** 2026-03-13

**Authors:** Nazaret Gamez, Abdulmunaim M Eid, Belen Pascual, Daling Li, Yanling Wang, Joseph C Masdeu, Stanley H Appel, Alireza Faridar

**Affiliations:** Stanley H. Appel Department of Neurology, Houston Methodist Neurological Institute, Houston, TX 77030, USA; Stanley H. Appel Department of Neurology, Houston Methodist Neurological Institute, Houston, TX 77030, USA; Stanley H. Appel Department of Neurology, Houston Methodist Neurological Institute, Houston, TX 77030, USA; Stanley H. Appel Department of Neurology, Houston Methodist Neurological Institute, Houston, TX 77030, USA; Stanley H. Appel Department of Neurology, Houston Methodist Neurological Institute, Houston, TX 77030, USA; Stanley H. Appel Department of Neurology, Houston Methodist Neurological Institute, Houston, TX 77030, USA; Stanley H. Appel Department of Neurology, Houston Methodist Neurological Institute, Houston, TX 77030, USA; Stanley H. Appel Department of Neurology, Houston Methodist Neurological Institute, Houston, TX 77030, USA

**Keywords:** frontotemporal dementia, Tregs, inflammation, immune system, cytokines

## Abstract

Frontotemporal dementia (FTD) encompasses a heterogenous and clinically diverse group of disorders, including the behavioural variant frontotemporal dementia, the non-fluent and the semantic variants of primary progressive aphasia. While these subtypes present with distinct clinical features, emerging evidence implicates neuroinflammation as a shared pathogenic mechanism. Nevertheless, little is known regarding the status of the peripheral immune system in the pathogenesis of FTD. Blood samples were obtained from 27 individuals with a clinical diagnosis of FTD (7 behavioural variants FTD, 10 non-fluent and 10 semantic variants of primary progressive aphasia) and 25 age-matched healthy controls. The immunophenotypes of peripheral immune cell populations were assessed with multicolour flow cytometry. Regulatory T cells were isolated and co-cultured with responder T cells and proliferation was determined by 3H-thymidine incorporation. The immune-related transcriptomic profile of isolated monocytes was analysed using the NanoString Human Inflammation Panel. Plasma levels of inflammatory cytokines and chemokines were quantified using the Olink® Target 48 Cytokine panel. Our analysis demonstrated that the suppressive function of regulatory T cells on responder T cells proliferation was significantly compromised in FTD individuals compared to healthy controls (*P* < 0.05). Transcriptomic profiling of FTD monocytes revealed a potential dysregulation of 153 immune-related genes. Enrichment analysis showed that these genes were mainly involved in chemokine-mediated signalling pathway, monocyte and lymphocyte chemotaxis, response to interferon-gamma and positive regulation of ERK1 and ERK2 cascades. Proteomic analysis of plasma inflammatory mediators showed a significant increase in the pro-inflammatory cytokine TNFa (*P* < 0.05) and the chemokines CXCL10, CCL3, CCL19, CSF1 (*P* < 0.05) and CXCL12 (*P* < 0.01) in FTD individuals compared to healthy controls. These findings provide the first evidence that the immunomodulatory function of regulatory T cells is compromised in individuals with FTD. In addition, there is a dysregulation of inflammation-related gene expression in peripheral monocytes and an increase of plasma inflammatory chemokines and cytokines in FTD individuals. Further investigation is warranted to assess the therapeutic potential of restoring dysfunctional regulatory T cells and modulating the inflammatory profile in the clinical setting of FTD.

## Teaser

Gamez et al., report impaired Treg function in frontotemporal dementia (FTD), accompanied by dysregulated inflammatory gene expression in monocytes and elevated plasma cytokines. These findings offer new insights into the systemic immune status in FTD and suggest that targeting inflammation holds therapeutic potential, warranting further investigation into immunomodulatory strategies.

## Graphical Abstract

**Graphical Abstract:**
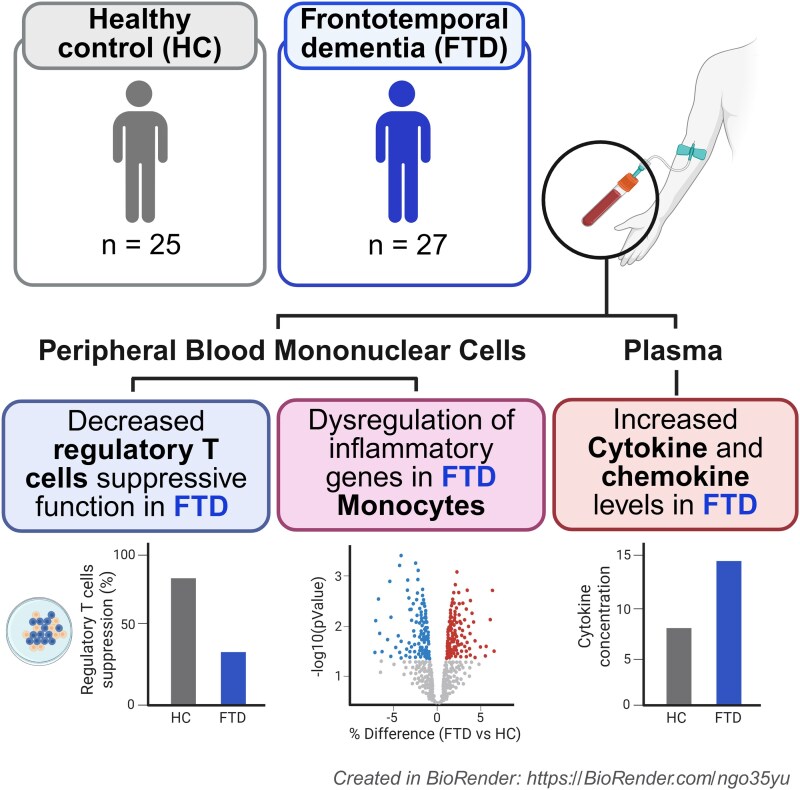

## Introduction

Frontotemporal dementia (FTD) encompasses a heterogenous and clinically diverse group of disorders,^1,2^ including the behavioural variant frontotemporal dementia (bvFTD^3^), as well as the non-fluent (nfvPPA) and the semantic variant (svPPA) of primary progressive aphasia.^4^ Although FTD subtypes present with distinct clinical and neuroanatomic features, converging evidence highlights neuroinflammation as a shared pathogenic mechanism.^5-9^ Neuroinflammation, characterized by the activation of glial cells (microglia and astrocytes) and increased secretion of inflammatory molecules, is a well-established hallmark across neurodegenerative diseases, including frontotemporal lobar degeneration (FTLD), Alzheimer’s disease (AD), amyotrophic lateral sclerosis (ALS) and Parkinson’s disease (PD).^10,11^ Growing evidence also suggests that, beyond glial cells, peripheral immune cells play a critical role in the pathology of neurodegenerative diseases.^12-14^ In neurodegenerative conditions, the integrity of the blood–brain barrier is often compromised, which along with the upregulation of chemoattractant signals, facilitate the recruitment and transmigration of peripheral immune populations into the central nervous system.^15^ In this context, the presence of T cells in the post-mortem brain of FTLD patients has been described,^16,17^ which contributes to neuronal loss in the pro-inflammatory milieu.^18^ Despite growing insights into glial-mediated mechanisms, little is known regarding the status of the systemic immune responses in the clinical setting of FTD. Given the increasing interest in immunomodulatory strategies for FTD, targeting systemic inflammation represents a promising therapeutic avenue to potentially slow or alter disease progression.^19,20^ A clearer understanding of the peripheral immune landscape in FTD is essential for the development of effective, mechanism-based interventions.

Systemic regulatory T lymphocytes (Tregs) are a specialized subset of T cells that are critically involved in suppressing inflammation, limiting immune activation and promoting tissue repair.^21,22^ Tregs also have been found to induce neuroprotective phenotypes in astrocytes^23^ and microglia.^24^ Indeed, Treg dysfunction has been associated with an enhanced pro-inflammatory phenotype in myeloid cells, ultimately leading to chronic and imbalanced immune responses.^21,25^ In neurodegenerative disorders, including AD, ALS and PD, we have previously identified that Tregs display impaired immunomodulatory functions, which correlate with a shift towards a pro-inflammatory response.^26-28^ In this study, we aim to assess for the first time the immunophenotype and functional status of Tregs in the clinical setting of FTD. In addition to T cells, circulating monocytes are key players in immune surveillance.^29^ Upon their recruitment into tissues, these cells perform essential functions, including phagocytosis, antigen presentation and the modulation of inflammation through the release of cytokines and other signalling molecules^30-33^. Dysregulation of the inflammatory response in circulating monocytes has been linked to the severity of numerous neurodegenerative disorders.^34-36^ In line with this, a previous report from our group highlighted the pro-inflammatory status of peripheral monocytes in AD dementia stage.^37^ In this study, in addition to analysing Tregs, we further investigate the peripheral inflammatory landscape in FTD by examining the inflammatory gene expression profiles of circulating monocytes and assessing plasma levels of cytokines and chemokines in individuals with FTD compared to age-matched healthy controls (HC). These findings will provide novel insights into the mechanisms driving inflammatory signatures in the clinical setting of FTD and suggest potential immunomodulatory targets.

## Material and methods

### Patient recruitment and clinical diagnosis

FTD individuals and age-matched HC were recruited into the study at the Houston Methodist Nantz National Alzheimer’s Center (NNAC) (Table 1 and Supplementary Table 1). Written consent was obtained from all participants following ethics approval from the Institutional Review Board at Houston Methodist Research Institute. Clinical, neuropsychiatric and imaging evaluations of 27 FTD individuals [bvFTD (*n* = 7), nfvPPA (*n* = 10) and svPPA (*n* = 10)], as well as for the age-matched HC group (*n* = 25) were performed and documented in the NNAC Frontotemporal Degeneration unit. The diagnosis of FTD was established based on international consensus criteria for bvFTD^3^ and for svPPA as well as nfvPPA.^4^ Alternative aetiologies were excluded based on extensive neuroimaging and biomarker assessments, including MRI, FDG-PET, amyloid PET, 18F-AV1451 and Flortaucipir PET scan. All patients had a negative amyloid PET scan, which ruled out the diagnosis of AD. Dementia severity was staged using the clinical dementia rating (CDR) scale^38,39^ that ranges from 0 (no cognitive impairment) to 3 (severe dementia). Individuals were considered HC based on clinical and neuropsychological screening (CDR = 0), and absence of neurological, psychiatric or other major medical illnesses. Furthermore, individuals with known or chronic inflammatory conditions or those receiving immunomodulatory or immunosuppressive medications were excluded from both the FTD and the HC cohorts.

**Table 1:** Demographic information of HC and FTD groups

Diagnosis	*N*	Age (years) Mean ± SD	Sex M/F	CDR Mean ± SD
**HC**	25	67.56 ± 9.798	10/15	0
**FTD**	27	64.11 ± 9.023	9/18	1.037 ± 0.8979
***P value***		*P* = 0.1924	*P* = 0.7743	*P* < 0.001 (HC < FTD)

M = male; F = female. Group comparisons using unpaired *t*-test (age and CDR). Chi-square test of independence (and Fisher’s exact test) to calculate *P* value between HC and FTD groups based on sex distribution (male versus female).

### Blood sample collection, processing and immune cell isolation

Single blood samples were collected from all participants (27 FTD and 25 HC) via phlebotomy using EDTA tubes. Briefly, each blood sample was diluted 1:1 with Dulbecco’s phosphate-buffered saline and centrifuged with brake off using Lymphoprep density gradient (SEMCELL Technologies). Plasma was then aliquoted and stored at −80°C for later analysis. Peripheral blood mononuclear cells (PBMCs) were isolated, and Tregs and Tresps were separated using the CD4+ CD25+ Regulatory T Cell Isolation Kit (Miltenyi Biotec) according to the manufacturer’s instructions. To enhance Treg purity, the positively selected CD4+ CD25+ Tregs fraction was passed twice through the MS column. The effluent solution containing CD4+ CD25− cells was collected as Tresps. Monocytes were isolated through negative selection using the Human Pan Monocyte Isolation Kit (Miltenyi Biotec).

### Flow cytometry

The immunophenotype of Tregs and immune cell population percentages of Tregs, Tresps, CD8T cells and natural killer cells (NKC) were analysed from peripheral blood of 17 FTD and 14 HC individuals by multicolour flow cytometric analyses. The following antibodies were utilized: anti-CD3 BV650 (BD Biosciences), anti-CD4 APC-H7 (BD Biosciences), anti-CD25 perCPCy5.5 (BD Biosciences), anti-CD8 BV450 (BD Biosciences), anti-CD16 PE-Cyanine7 (eBioscience), anti-CD127 PE (Invitrogen) and anti-CD56 APC (eBioscience). For intracellular staining of Foxp3, cells were fixed and permeabilized using FoxP3/Transcription Factor Staining Buffer Set (eBioscience) following staining with anti-Foxp3 Alexa Fluor 488 antibody (eBioscience). The Live/Dead® Fixable Blue Dead Cell Stain Kit (Invitrogen) was used to stain dead cells. Fluorescence-minus-one (FMO) controls were used to identify gating boundaries, and IgG isotype controls (eBioscience) to evaluate the background signal. Stained cells were acquired on a BD Bioscience LSR II bench top flow cytometer at the Flow Cytometry Core in Houston Methodist Research Institute. Data were analysed using the BD FACSDIVA software and following the gating strategy to identify the immune cell populations as previously described.^26-28^

### Treg suppression assay on Tresp proliferation

Isolated Tregs and Tresps from the same individual (*n* = 21 FTD and 12 HC) were co-cultured at a 1:1 ratio in a 96-well plate for five consecutive days in at least triplicates. A CD3/CD28 T cell stimulation reagent (Multenyi Biotec) that specifically induces the proliferation of Tresps was added to the co-culture to evaluate the Treg suppressive function of Tresps proliferation. Following 5 days of co-culture, 3H-thymidine was added to the medium (1 Ci/well; Amersham Life Sciences) for 18 h. Then, cells were harvested, and 3H-thymidine uptake was assessed by a gas-operated-plate reader (Packard Instruments) to evaluate the proliferation of Tresps via 3H-thymidine incorporation. Treg suppressive function was calculated as % suppression = 100—[(counts per minute of Tresp proliferation in co-culture with Tregs/counts per minute of Tresp proliferation in the absence of Tregs)×100], as previously reported.^26-28^

### Monocyte RNA purification and Nanostring analysis

Monocytes were isolated from the peripheral blood of a cohort of 11 FTD and 8 HC individuals. RNA was purified from monocytes using Trizol and the Direct-zol RNA MiniPrep Kit (Zymo Research), following the manufacturer’s instructions. Nanostring experiments were performed at the Genomic and RNA Profiling Core of Baylor College of Medicine. The Nanostring nCounter Human Inflammation Panel, comprising 770 human inflammation-related transcripts with 15 internal reference genes, was used to profile monocyte RNA transcripts. Data normalization was carried out with the nSolver analysis software. Then, data were transformed to logs and statistical analysis were performed using GraphPad Prims software version 9.0. Statistical comparisons between groups were conducted using unpaired *t*-tests and setting the threshold for significance at *P*-value < 0.05. Given that the analysis focused on a hypothesis-driven gene set (predefined set of 770 immune-related genes), involved a small sample size (11 FTD versus 8 HC) and was exploratory in nature, no multiple-testing correction was applied. Differential gene expression results were visualized using a volcano plot generated using open-source R packages. Genes were plotted with percent difference (FTD versus HC) on the x-axis and -log10 (*P*-value) on the y-axis. Threshold for statistical significance was defined as *P*-value < 0.05. Significant genes were colour-coded to distinguish upregulated (red) and downregulated (blue) transcripts.

### STRING and Cytoscape analysis

Further analysis of monocyte inflammation-related transcripts data was conducted using the STRING database (https://string-db.org/). Briefly, the list of significantly upregulated and downregulated genes identified in FTD monocytes was uploaded to STRING to analyse protein–protein interaction (PPI) networks and to perform Gene Ontology (GO) enrichment analysis. The analysis included the identification of significantly enriched biological processes, molecular functions and cellular components associated with the differentially expressed gene set. The confidence score was set at 0.7 (high confidence). Next, the STRING PPI network was exported to Cytoscape 3.10.3 where protein nodes were coloured based on the percentage difference (FTD versus HC).

### Plasma proteomic analysis

Inflammatory mediators were assessed in plasma samples (*n* = 14 FTD and 16 HC) using the Olink® Target 48 Cytokine Panel (Olink Proteomics) and the Proximity Extension Assay technology. This technique enables the simultaneous quantification of 45 different cytokines and chemokines by using paired antibodies linked to a complementary oligonucleotide sequence that can be then detected by next-generation sequencing on the NovaSeq 6000 Sequencing System.

### Statistical analysis

All statistical analyses were performed using GraphPad Prism software version 9.0 (GraphPad Software, Inc). Normality of the data was assessed using the D’Agostino-Pearson omnibus test for the comparison FTD versus HC or the Shapiro–Wilk test when comparing the FTD subtypes versus HC. Comparison among the FTD and HC groups were assessed using either unpaired Student’s *t*-test or the nonparametric Mann–Whitney test. One-way ANOVA followed by Dunnet’s multiple comparison test or the nonparametric Kruskal–Wallis test followed by Dunn’s to correct for multiple comparisons were used when assessing differences between each of the FTD subtypes (bvFTD, nfvPPA and svPPA) versus HC. Data were expressed as mean ± SEM, and statistical significance was set at *P* values < 0.05 (*P < 0.05, **P < 0.01, ***P < 0.001). Differential expression analysis of inflammation-related transcripts in monocytes was conducted using multiple unpaired *t*-tests assuming a single pooled variance (FTD versus HC). A threshold of *P*-value <0.05 was applied to statistically significant differences.

## Results

### The suppressive function of Tregs is compromised in FTD patients

**Figure 1:**
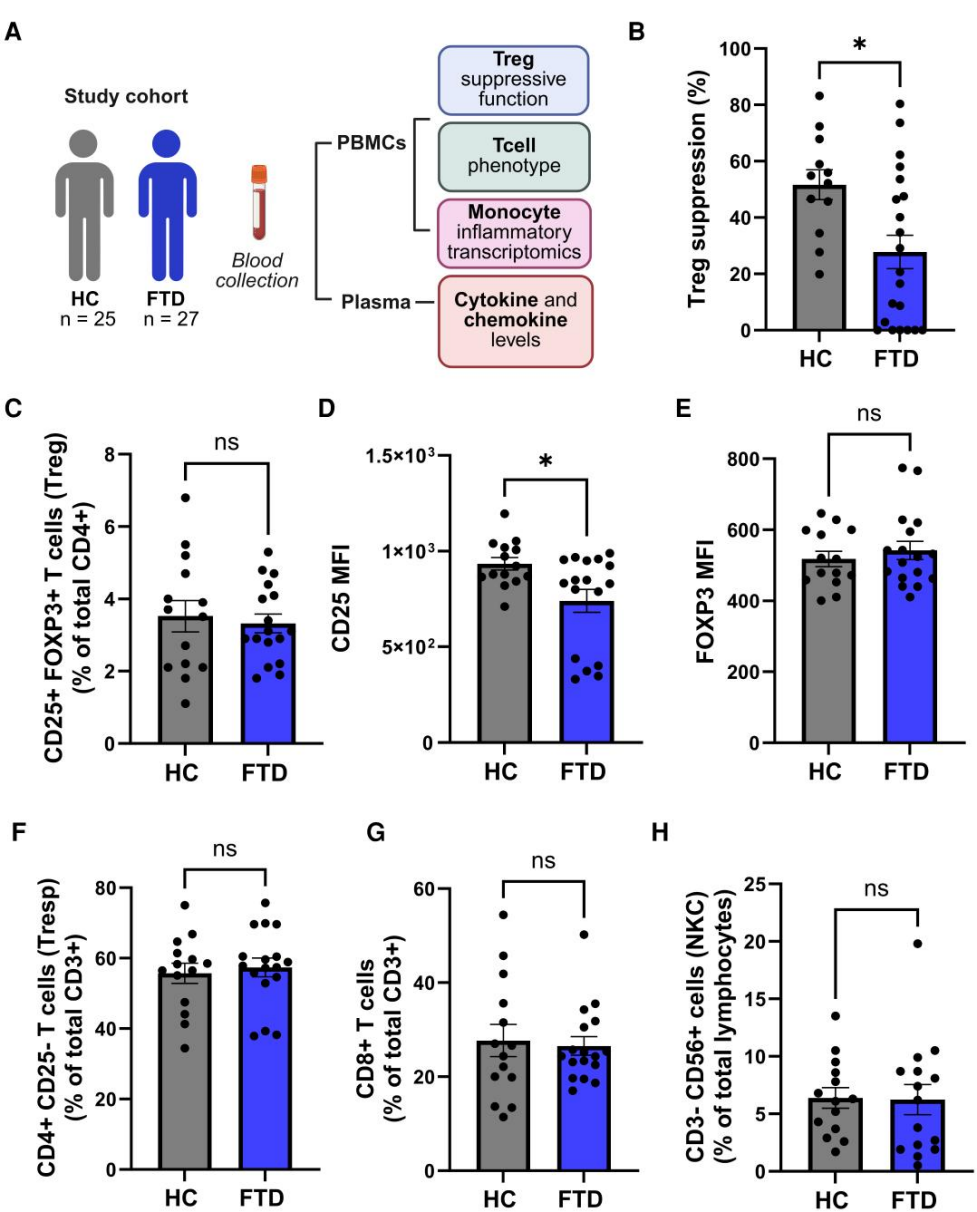
**The Treg immunomodulatory function is compromised in FTD.** (A) Study design to assess Treg suppressive function, Tcell immunophenotype, monocyte’s inflammatory profile and plasma cytokine and chemokine levels in HC and FTD. Created in BioRender: https://BioRender.com/1b4xmui  **(B)** The Treg suppression of Tresp proliferation was significantly reduced in the FTD group compared to HC. (**C)** No changes with respect to the number of Tregs were observed among the groups. (**D)** CD25 mean fluorescent intensity (MFI) in Tregs of FTD individuals was significantly decreased compared to HC. (**E)** FOXP3 MFI in Tregs did not exhibit significant changes in FTD individuals compared to HC. The number of Tresp **(F)**, CD8 T cell **(G)**, or natural killer (NK) cells **(H)** did not differ among the FTD and HC groups. Graphs depicting mean ± SEM; unpaired Student *t*-test (B-F), or nonparametric Mann–Whitney test (**G**, **H**), *P < 0.05, ns & non-significant; Each individual data point represents a biological replicate; *n* = 12 HC, 21 FTD in **B**, and *n* = 14 HC, 17 FTD in C-H.

In this study (Fig. 1 (A)), a total of 25 cognitively unimpaired HC individuals and 27 individuals with clinical diagnoses associated with FTD (7 bvFTD, 10 nfvPPA, 10 svPPA) were recruited (Table 1 and Supplementary Table 1). Blood samples were collected to obtain PBMCs and plasma. PBMCs were used to isolate Tregs and Tresps for assessing the suppressive function of Tregs, as well as to isolate monocytes for evaluating their inflammatory gene profile. Due to technical limitations and sample availability, not all individuals were assessed using all experimental approaches (Supplementary Table 2). Specifically, Treg dysfunction was analysed in 12 HC and 21 FTD patients, while the flow cytometric analysis of T cells phenotype was evaluated in 14 HC and 17 FTD patients. The inflammatory transcriptomic profiling of monocytes was performed in 8 HC and 11 FTD. Additionally, plasma samples of 16 HC and 14 FTD were analysed to measure cytokine and chemokine levels.

CD4+ CD25+ Tregs and CD4+ CD25-Tresps were isolated from PBMCs from a cohort of 21 FTD patients and 12 age-matched HC individuals. The Treg suppressive function (%) on Tresp proliferation in FTD patients was significantly reduced compared to HC (1:1 Treg:Tresp ratio: FTD = 27.81 ± 5.917%, versus HC = 51.65 ± 5.302%, *P* = 0.0111) (Fig. 1 (B)). Exploratory analyses by FTD subtype likely suggest that Treg suppressive dysfunction may be more pronounced in svPPA (Supplementary Fig. 1), but larger studies are needed to confirm subtype-specific differences. Flow cytometric analyses were performed to assess immune cell population percentages and the phenotypic characteristics of Tregs in blood samples of FTD compared to HC. The number of Tregs (identified as CD4^+^ CD25^+^ Foxp3^+^) was comparable among the FTD and the HC groups (HC = 3.521 ± 0.4358% versus FTD = 3.318 ± 0.2588%; *P* = 0.6787) (Fig. 1 (C)). Remarkably, a significant decline was identified in the mean fluorescent intensity (MFI) of CD25 in the Treg population of FTD individuals when compared to the HC group (HC = 934.1 ± 32.67 versus FTD = 740.4 ± 60.54; *P* = 0.0130) (Fig. 1 (D)), which appears to be primarily driven by the nfvPPA and the svPPA subtypes (*P* < 0.01) (Supplementary Fig. 1 C). No differences were detected between the FTD and the HC groups when analysing the MFI of Foxp3 in the Treg population (Fig. 1 (E)). The percentages of Tresps (CD4+ CD25-) (Fig. 1 (F)), CD8 T cells (Fig. 1 (G)) and CD8-CD56+ NKC (Fig. 1 (H)) were comparable among the FTD and HC groups.

### The transcriptomic profile of peripheral monocytes is highly pro-inflammatory in FTD patients

**Figure 2:**
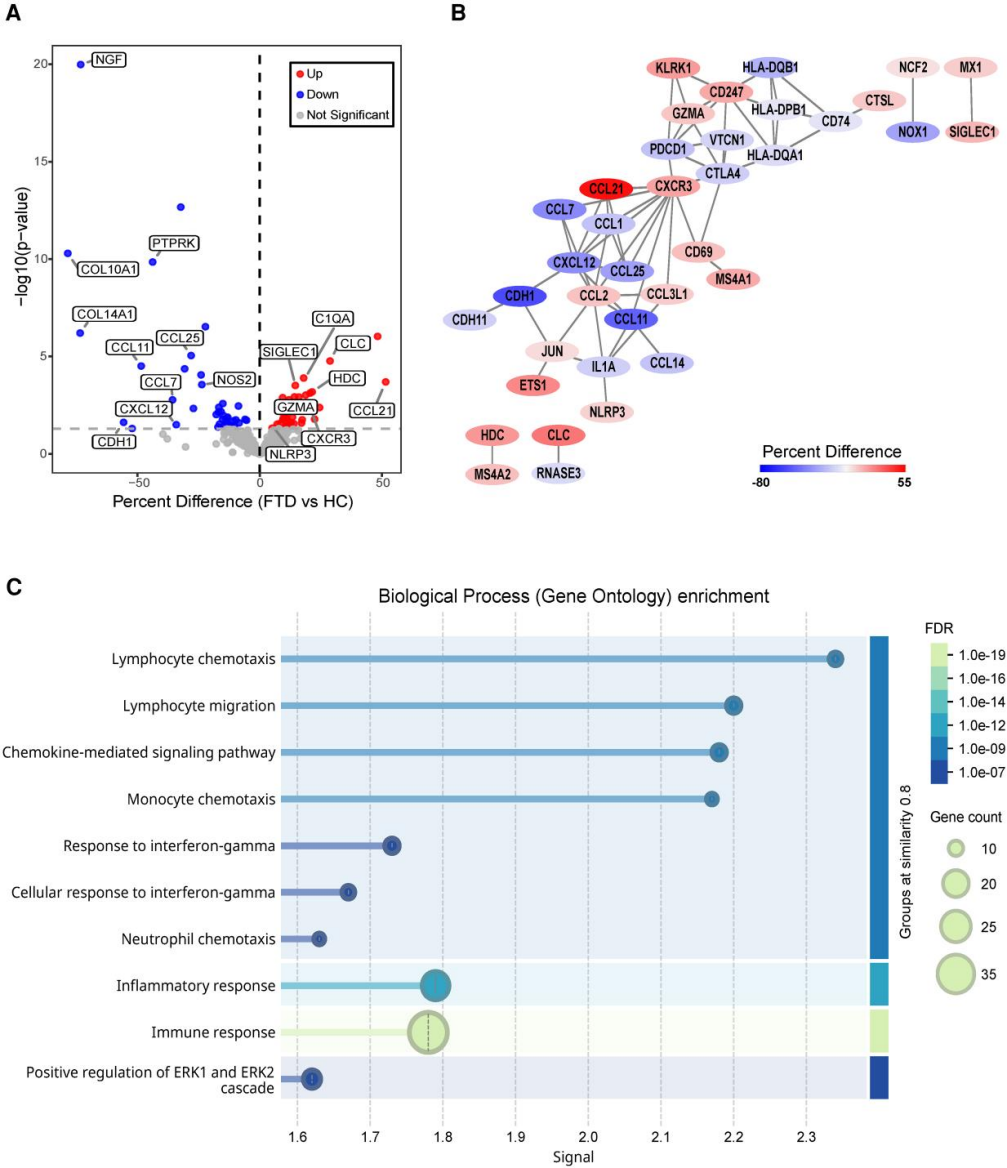
**Dysregulation of monocyte’s immune related genes in FTD. (A)** Volcano plot displaying -log10(*P*-value) on the y-axis versus percent difference (FTD versus HC) on the x-axis from the differential expression analysis of inflammation-related transcripts in monocytes. Out of all 770 transcripts analysed, 37 were significantly upregulated (red) and 40 were significantly downregulated (blue) in FTD compared to HC. The horizontal dashed line represents the significant threshold (*P* < 0.05). Some of the significantly altered genes are labelled. **(B)** PPI network of significantly dysregulated inflammation-related genes in FTD monocytes defined by the STRING software and visualized within Cytoscape. Nodes (circles) represent proteins whose transcripts were found to be significantly dysregulated in FTD monocytes compared to HC. Each protein node was colour-coded based on the value of percent difference (FTD versus HC) to indicate upregulation (red) or downregulation (blue). The edges (lines) indicate both physical and functional interactions between proteins. **(C)** GO enrichment of the overrepresented pathways dysregulated in FTD monocytes. Signal is defined as a weighted harmonic mean between the observed/expected ration and -log(FDR). False discovery rate describes how significant the enrichment is. Shown are *P*-values corrected for multiple testing within each category using the Benjamini–Hochberg procedure. *n* = 8 HC, 11 FTD.

The inflammatory profile of peripheral monocytes was determined by analysing their isolated RNA using a NanoString gene expression panel of 770 human immune factors. Out of all 770 inflammation-related transcripts analysed, 37 were observed to be significantly upregulated while 40 were significantly downregulated in FTD patients compared to HC (Fig. 2 (A) and Supplementary Tables 3 and 4). Remarkably, FTD monocytes exhibited significantly increased expression of pro-inflammatory genes, including NLR family pyrin domain containing 3 (*NLRP3),* C-X-C Motif Chemokine Receptor 3 (*CXCR3),* Complement C1qA *(C1QA)* and CC motif chemokine ligand 21 *(CCL21).* Nerve growth factor (*NGF)* and the Treg and Th2 chemoattractant *CCL1*, as well as other chemokines including *CCL7*, *CCL11* and *CCL25* were found to be downregulated in FTD monocytes (Fig. 2 (A)). However, as multiple-comparison correction was not applied, these findings warrant further validation. Then, PPI analyses were performed in the STRING database and visualized within Cytoscape to identify key pathways and molecular interactions that are dysregulated in FTD-monocytes. The enriched network was centred on chemokine-signalling (CXCR3) but also involved the NLRP3 inflammasome protein and granzyme A (GZMA) that participates in cytotoxic cell-mediated apoptosis (Fig. 2 (B)). Moreover, the transcription factors JUN and ETS1 involved in regulating gene expression in immune response were also identified in the PPI analysis (Fig. 2 (B)). Next, to identify the biological processes and molecular functions that are dysregulated in circulating monocytes of FTD compared to HC, we conducted GO analysis with an adjusted *P* value <0.05. The top 10 dysregulated pathways in FTD-monocytes were mainly involved in lymphocyte chemotaxis, lymphocyte migration, chemokine-mediated signalling pathway, monocyte chemotaxis, response to interferon gamma, neutrophil chemotaxis, inflammatory/immune response and positive regulation of ERK1 and ERK2 cascade (Fig. 2 (C)).

### Pro-inflammatory cytokines are increased in the plasma of FTD individuals

**Figure 3:**
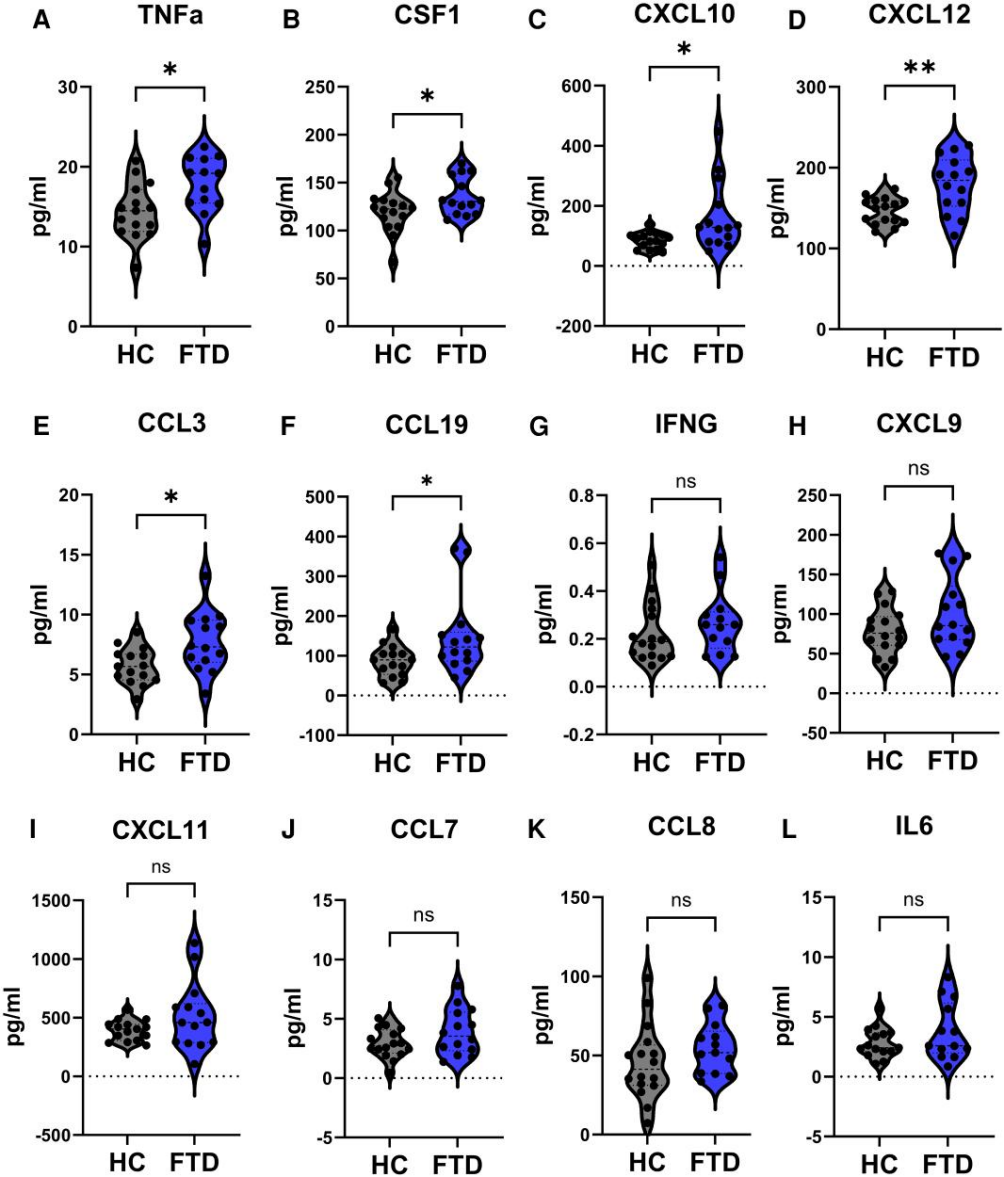
**Levels of inflammatory plasma markers in FTD and HC.** The concentration of 45 inflammatory cytokines and chemokines was measured in the plasma of HC and FTD individuals. The levels of TNF **(A),** CSF1 **(B),** CXCL10 **(C),** CXCL12 **(D)**, CCL3 **(E) and** CCL19 **(F)** were significantly elevated in FTD plasma compared to HC. Levels of interferon gamma **(G)** CXCL9 **(H)**, CXCL11 **(I),** CCL7 **(J),** CCL8 **(K)** and IL6 **(L)** exhibited a trend towards an increase in plasma of FTD versus HC. Graphs depicting mean ± SEM; Unpaired Student *t*-test (**A**, **B**, **D**, **E**, **G-L**), or nonparametric Mann–Whitney test (**C** and **F**), *P < 0.05, **P < 0.01, ns = non-significant. Each individual data point represents a biological replicate; *n* = 16 HC, 14 FTD.

Plasma levels of cytokines and chemokines were measured in individuals with FTD and HC participants using the Olink® Target 48 Cytokine Panel. Out of the 45 inflammatory markers analysed, 8 protein measurements fell below the lower limit of detection and were excluded from the analysis (IL-33, IL-2, IL-1B, IL-4, TSLP, IL-17F, IL-13 and CSF2). Five inflammatory mediators were found to be significantly increased in FTD individuals. These included the pro-inflammatory cytokines tumour necrosis factor alpha (TNFa) and colony-stimulating factor-1 (CSF1) (Fig. 3 (A, B)) as well as the chemokines CXCL10, CXCL12, CCL3 and CCL19 (Fig. 3 (C-F)), which levels significantly increased in the plasma of FTD patients compared to HC. Although not statistically significant, several additional cytokines and chemokines exhibited a trend towards elevated levels in FTD plasma, including interferon gamma, CXCL9, CXCL11, CCL7, CCL8 and the interleukin IL6 (Fig. 3 (G-L)). There were no significant differences among the remaining inflammatory mediators (Supplementary Fig. 2). Although differences between FTD subtypes and HC (Supplementary Fig. 3) were less pronounced than those observed when considering FTD as a whole, the limited sample size within each subgroup warrants cautious interpretation and highlights the need for larger, subtype-focused studies.

## Discussion

Tregs are characterized by a high expression of the transcription factor Foxp3 and the surface molecule CD25.^21,40^ Reduction in Treg numbers, decreased expression of Foxp3 or CD25 and defects in their suppressive activity have been described in neurodegenerative diseases.^26-28^ Our study demonstrates for the first time that Treg suppressive function on Tresp proliferation is compromised in FTD patients. Although the counts of Tregs were comparable among HC and the FTD subtypes, FTD Tregs displayed a significant reduction in CD25 MFI, suggesting phenotypic alterations that may underly the observed functional impairment. CD25 is essential for Treg homeostasis, survival and suppressive function.^41,42^ Notably, Tregs with reduced CD25 expression exhibit a partial suppressive function in autoimmune disorders.^43-47^ The loss of function of Tregs in FTD patients might be associated with enhanced neuroinflammation and disease worsening. To support this hypothesis, *in vivo* studies using animal models provide compelling evidence for the involvement of Tregs in neurodegeneration. In a murine model of AD, the depletion of Tregs led to an upregulation of genes associated with astrogliosis and microgliosis, accompanied by an increased proteinopathy in the hippocampus.^48,49^ Two potential mechanisms may explain how compromised Tregs contribute to enhanced neuroinflammation and neurodegenerative pathology. First, peripheral Tregs regulate the inflammatory priming of adaptive immune cells that subsequently migrate to the brain. When Treg suppressive function is impaired, pro-inflammatory immune cells infiltrate the central nervous system (CNS) and exacerbate neuroinflammation and disease progression. Second, Tregs can also directly migrate to the brain, where they will maintain glial homeostasis by modulating microglial and astrocytic reactivity.^22^ Functionally impaired Tregs will not be able to sustain microgliosis and astrogliosis in response to neuropathological insults, which results in the exacerbation of neuroinflammation. Accordingly, the restoration and expansion of Tregs in various models of neurodegeneration have been shown to promote a neuroprotective microglial phenotype, suppress neuroinflammation and attenuate disease progression.^50^ In a PD mouse model, transplantation of activated Tregs reduced dopaminergic neuronal loss, diminished microglial neurotoxicity and elevated the levels of neurotrophic factors such as BDNF and GDNF.^51^ Similarly, in ALS models, Tregs promoted a neuroprotective phenotype in microglia.^52^ These data suggest that our finding of dysfunctional Tregs in the clinical setting of FTD could lead to therapeutic strategies restoring Tregs, thus modulating the neuroinflammatory response and supporting neuronal survival.

We further assessed the status of the peripheral innate immune system by examining the profile of isolated monocytes in FTD patients. Our analysis revealed a remarkable dysregulation of immune-related genes in FTD monocytes, characterized by increased expression of pro-inflammatory genes such as *NLRP3, CXCR3, C1QA* and *CCL21*, and downregulation of nerve growth factor (*NGF*), the Treg/Th2 chemoattractant *CCL1*, as well as other chemokines including *CCL7*, *CCL11* and *CCL25*. A pattern of increased expression of known pro-inflammatory genes alongside reduced levels of certain chemokines has previously been reported in chronically activated monocytes exhibiting features of exhaustion.^53-55^ The distinct inflammatory feature of circulating FTD-monocytes may be linked to the impaired suppressive function exhibited by their Tregs. Supporting this notion, previous studies have shown that Tregs with improved immunomodulatory function can drive monocyte differentiation towards an anti-inflammatory phenotype, marked by reduced production of pro-inflammatory cytokines and elevated levels of IL-10.^56^ Moreover, activated pro-inflammatory monocytes can promote Treg dysfunction, while impaired Tregs fail to suppress pro-inflammatory myeloid cells.^27^ Thus, the pro-inflammatory state of monocytes along with Treg dysfunction appear to be mutually reinforcing processes, suggesting a bidirectional relationship in the context of neurodegenerative diseases. Compelling reports have linked the existence of proinflammatory monocytes in the periphery with neurodegeneration.^57^ Among the mechanisms, pro-inflammatory monocytes likely contribute to the sustained production of inflammatory mediators in the brain, leading to the loss of microglial protective function and ultimately promoting disease progression and cognitive decline.^58,59^ Nevertheless, the role of infiltrating monocytes on neurodegeneration remains controversial as monocyte-derived macrophages in the brain can ameliorate AD progression by acting as reinforcements when microglia are unable to contain the damage.^60^ More studies are required to unravel the role of these circulating pro-inflammatory monocytes, their brain infiltration mechanisms and their interactions with microglia in FTD disease progression.

Finally, our data indicate increased levels of plasma pro-inflammatory cytokines and chemokines in FTD individuals, further supporting the evidence of peripheral inflammatory reaction in this disease. In agreement with recent studies,^8,61^ our findings showed that the concentration of pro-inflammatory cytokines, including TNF-α, CSF1, CXCL10, CXCL12, CCL3 and CCL19 were elevated in the plasma of FTD cases compared to HC. TNF-α is a central pro-inflammatory cytokine that orchestrates the initiation and regulation of the cytokine cascade during an inflammatory response.^62^ In a large cohort of autosomal dominant FTD individuals, higher baseline plasma levels of TNF were correlated with faster longitudinal cognitive and functional decline.^63^ Moreover, the inhibition of TNF-α signalling in the PS19 transgenic mouse model of FTLD attenuated disease pathology by reducing microgliosis and neuronal loss.^64^ In addition to TNFa, we observed increased plasma levels of CXCL10 and trends towards elevated levels of plasma CXCL9 and CXCL11 in FTD individuals, accompanied by increased expression of their shared receptor, CXCR3, in peripheral monocytes. The CXCL9-11/CXCR3 axis comprises a key immune cascade that mediates the migration of immune cells into the inflammation sites.^65^ This axis may facilitate the infiltration of T cells into the CNS, contributing to neurodegeneration pathology progression. Consistently, a three-dimensional human neuroimmune axis model demonstrated that the CXCL10/CXCR3 pathway regulates the infiltration of CD8+ T cells into AD cultures, leading to increased microglial activation, neuroinflammation and neurodegeneration.^66^ Furthermore, blockade of this cascade improved microglial function, reduced proteinopathy and alleviated neurodegeneration pathology.^67^ More importantly, growing evidence suggests that T cell recruitment into the brain and their interactions with glial cells have detrimental effects in neurodegeneration pathology in preclinical^18^ and clinical stages.^68,69^ Collectively, these findings highlight TNFa and CXCL9-11/CXCR3 pathways as potential therapeutic targets for modulating neuroinflammation and disease progression. Therefore, beyond using peripheral inflammatory signatures as biomarkers to assess neuroinflammation and facilitate patient stratification in clinical trials, these altered inflammatory cascades may be implicated in the pathogenesis of FTD, highlighting potential therapeutic targets for further investigation.

Our study has limitations. First, the sample size is relatively small, which is an inherent challenge when studying rare neurodegenerative syndromes such as FTD. Second, not all participants were tested for every assay reported, as the limited amount of blood available from each individual constrained the number of analyses that could be performed. Third, the diagnoses in our cohort are based on clinical criteria rather than histopathological confirmation, as a large number of participants are currently living and therefore post-mortem validation is not possible. Despite these limitations, our study provides important insights into peripheral immune alterations in FTD and identifies avenues for future research, particularly those aimed at restoring Treg function and targeting systemic inflammation. Moreover, although clinical rather than histopathological diagnosis can be viewed as a constraint, we consider the use of antemortem samples to be a major strength. Understanding biological changes during life is essential for advancing disease pathobiology, developing biomarkers and ultimately informing therapeutic interventions.

In conclusion, our study has revealed evidence of systemic immune dysregulation in FTD, supported by (i) a compromised suppressive function of Tregs, (ii) a pro-inflammatory signature of circulating monocytes and (iii) an increase in the concentration of plasma inflammatory cytokines and chemokines. Understanding the status of the peripheral immune system is a crucial step in elucidating the role of these immune cell populations in neurodegeneration. Further mechanistic studies are essential to elucidate the interplay between the peripheral and central immune systems in FTD and related diseases, as well as to determine whether targeting the peripheral immune dysregulation represents a meaningful therapeutic strategy.

## Supplementary Material

fcag089_Supplementary_Data

## Data Availability

The datasets generated in this study are included within the article. Raw data generated will be available upon reasonable request.

## References

[1] Irwin DJ, Cairns NJ, Grossman M, et al Frontotemporal lobar degeneration: Defining phenotypic diversity through personalized medicine. Acta Neuropathol. 2015;129(4):469–491. 25549971 10.1007/s00401-014-1380-1 PMC4369168

[2] Grossman M, Seeley WW, Boxer AL, et al Frontotemporal lobar degeneration. Nat Rev Dis Primers. 2023;9(1):40. 37563165 10.1038/s41572-023-00447-0

[3] Rascovsky K, Hodges JR, Knopman D, et al Sensitivity of revised diagnostic criteria for the behavioural variant of frontotemporal dementia. Brain. 2011;134(9):2456–2477. 21810890 10.1093/brain/awr179 PMC3170532

[4] Gorno-Tempini ML, Hillis AE, Weintraub S, et al Classification of primary progressive aphasia and its variants. Neurology. 2011;76(11):1006–1014. 21325651 10.1212/WNL.0b013e31821103e6 PMC3059138

[5] Bright F, Werry EL, Dobson-Stone C, et al Neuroinflammation in frontotemporal dementia. Nat Rev Neurol. 2019;15(9):540–555. 31324897 10.1038/s41582-019-0231-z

[6] Woollacott IOC, Toomey CE, Strand C, et al Microglial burden, activation and dystrophy patterns in frontotemporal lobar degeneration. J Neuroinflammation. 2020;17(1):1–27. 32778130 10.1186/s12974-020-01907-0 PMC7418403

[7] Bevan-Jones WR, Cope TE, Jones PS, et al Neuroinflammation and protein aggregation co-localize across the frontotemporal dementia spectrum. Brain. 2020;143(3):1010–1026. 32179883 10.1093/brain/awaa033 PMC7089669

[8] Malpetti M, Swann P, Tsvetanov KA, et al Blood inflammation relates to neuroinflammation and survival in frontotemporal lobar degeneration. Brain. 2025;148(2):493–505. 39155063 10.1093/brain/awae269 PMC7617268

[9] Pascual B, Funk Q, Zanotti-Fregonara P, et al Neuroinflammation is highest in areas of disease progression in semantic dementia. Brain. 2021;144(5):1565–1575. 33824991 10.1093/brain/awab057 PMC13016666

[10] Heneka MT, van der Flier WM, Jessen F, et al Neuroinflammation in Alzheimer disease. Nat Rev Immunol. 2025;25(5):321–352. 39653749 10.1038/s41577-024-01104-7 PMC13148177

[11] Ransohoff RM . How neuroinflammation contributes to neurodegeneration. Science (1979). 2016;353(6301):777–783. 10.1126/science.aag2590 27540165

[12] Bettcher BM, Tansey MG, Dorothée G, Heneka MT. Peripheral and central immune system crosstalk in Alzheimer disease—A research prospectus. Nat Rev Neurol. 2021;17(11):689–701. 34522039 10.1038/s41582-021-00549-x PMC8439173

[13] Haage V, De Jager PL. Neuroimmune contributions to Alzheimer’s disease: A focus on human data. Mol Psychiatry. 2022;27(8):3164–3181. 35668160 10.1038/s41380-022-01637-0 PMC9168642

[14] Prinz M, Priller J. The role of peripheral immune cells in the CNS in steady state and disease. Nat Neurosci. 2017;20(2):136–144. 28092660 10.1038/nn.4475

[15] Sweeney MD, Sagare AP, Zlokovic BV. Blood–brain barrier breakdown in Alzheimer disease and other neurodegenerative disorders. Nat Rev Neurol. 2018;14(3):133–150. 29377008 10.1038/nrneurol.2017.188 PMC5829048

[16] Hartnell IJ, Blum D, Nicoll JAR, Dorothee G, Boche D. Glial cells and adaptive immunity in frontotemporal dementia with tau pathology. Brain. 2021;144(3):724–745. 33527991 10.1093/brain/awaa457

[17] Hartnell IJ, Woodhouse D, Jasper W, et al Glial reactivity and T cell infiltration in frontotemporal lobar degeneration with tau pathology. Brain. 2024;147(2):590–606. 37703311 10.1093/brain/awad309 PMC10834257

[18] Chen X, Firulyova M, Manis M, et al Microglia-mediated T cell infiltration drives neurodegeneration in tauopathy. Nature. 2023;615(7953):668–677. 36890231 10.1038/s41586-023-05788-0 PMC10258627

[19] Neylan KD, Miller BL. New approaches to the treatment of frontotemporal dementia. Neurotherapeutics. 2023;20(4):1055–1065. 37157041 10.1007/s13311-023-01380-6 PMC10457270

[20] Self WK, Holtzman DM. Emerging diagnostics and therapeutics for Alzheimer disease. Nat Med. 2023;29(9):2187–2199. 37667136 10.1038/s41591-023-02505-2

[21] Sakaguchi S, Mikami N, Wing JB, Tanaka A, Ichiyama K, Ohkura N. Regulatory T cells and human disease. Annu Rev Immunol. 2020;38:541–566. 32017635 10.1146/annurev-immunol-042718-041717

[22] Liston A, Pasciuto E, Fitzgerald DC, Yshii L. Brain regulatory T cells. Nat Rev Immunol. 2024;24(5):326–337. 38040953 10.1038/s41577-023-00960-z

[23] Stym-Popper G, Matta K, Chaigneau T, et al Regulatory T cells decrease C3-positive reactive astrocytes in Alzheimer-like pathology. J Neuroinflammation. 2023;20(1):1–17. 36890536 10.1186/s12974-023-02702-3 PMC9996941

[24] Shi L, Sun Z, Su W, et al Treg cell-derived osteopontin promotes microglia-mediated white matter repair after ischemic stroke. Immunity. 2021;54(7):1527–1542.e8. 34015256 10.1016/j.immuni.2021.04.022 PMC8282725

[25] Machhi J, Kevadiya BD, Muhammad IK, et al Harnessing regulatory T cell neuroprotective activities for treatment of neurodegenerative disorders. Mol Neurodegener. 2020;15(1):32. 32503641 10.1186/s13024-020-00375-7 PMC7275301

[26] Beers DR, Zhao W, Wang J, et al ALS patients’ regulatory T lymphocytes are dysfunctional, and correlate with disease progression rate and severity. JCI Insight. 2017;2(5):e89530. 28289705 10.1172/jci.insight.89530 PMC5333967

[27] Faridar A, Thome AD, Zhao W, et al Restoring regulatory T-cell dysfunction in Alzheimer’s disease through ex vivo expansion. Brain Commun. 2020;2(2):fcaa112. 32954348 10.1093/braincomms/fcaa112 PMC7472911

[28] Thome AD, Atassi F, Wang J, et al Ex vivo expansion of dysfunctional regulatory T lymphocytes restores suppressive function in Parkinson’s disease. NPJ Parkinsons Dis. 2021;7(1):41. 33986285 10.1038/s41531-021-00188-5 PMC8119976

[29] Schultze JL, Mass E, Schlitzer A. Emerging principles in myelopoiesis at homeostasis and during infection and inflammation. Immunity. 2019;50(2):288–301. 30784577 10.1016/j.immuni.2019.01.019

[30] Becher B, Tugues S, Greter M. GM-CSF: From growth factor to central mediator of tissue inflammation. Immunity. 2016;45(5):963–973. 27851925 10.1016/j.immuni.2016.10.026

[31] Jakubzick CV, Randolph GJ, Henson PM. Monocyte differentiation and antigen-presenting functions. Nat Rev Immunol. 2017;17(6):349–362. 28436425 10.1038/nri.2017.28

[32] Amorim A, De Feo D, Friebel E, et al IFNγ and GM-CSF control complementary differentiation programs in the monocyte-to-phagocyte transition during neuroinflammation. Nat Immunol. 2022;23(2):217–228. 35102344 10.1038/s41590-021-01117-7

[33] Menezes S, Melandri D, Anselmi G, et al The heterogeneity of Ly6Chi monocytes controls their differentiation into iNOS + macrophages or monocyte-derived dendritic cells. Immunity. 2016;45(6):1205–1218. 28002729 10.1016/j.immuni.2016.12.001 PMC5196026

[34] Grozdanov V, Bliederhaeuser C, Ruf WP, et al Inflammatory dysregulation of blood monocytes in Parkinson’s disease patients. Acta Neuropathol. 2014;128(5):651. 25284487 10.1007/s00401-014-1345-4 PMC4201759

[35] Munawara U, Catanzaro M, Xu W, et al Hyperactivation of monocytes and macrophages in MCI patients contributes to the progression of Alzheimer’s disease. Immun Ageing. 2021;18(1):1–25. 34154615 10.1186/s12979-021-00236-x PMC8215492

[36] Zhao W, Beers DR, Hooten KG, et al Characterization of gene expression phenotype in amyotrophic lateral sclerosis monocytes. JAMA Neurol. 2017;74(6):677–685. 28437540 10.1001/jamaneurol.2017.0357 PMC5822209

[37] Thome AD, Faridar A, Beers DR, et al Functional alterations of myeloid cells during the course of Alzheimer’s disease. Mol Neurodegener. 2018;13(1):61. 30424785 10.1186/s13024-018-0293-1 PMC6233576

[38] Morris JC . The clinical dementia rating (CDR): Current version and scoring rules. Neurology. 1993;43(11):2412–2414. 10.1212/wnl.43.11.2412-a 8232972

[39] O’Bryant SE, Waring SC, Cullum CM, et al Staging dementia using clinical dementia rating scale sum of boxes scores: A Texas Alzheimer’s research consortium study. Arch Neurol. 2008;65(8):1091–1095. 18695059 10.1001/archneur.65.8.1091 PMC3409562

[40] Sakaguchi S . Naturally arising Foxp3-expressing CD25 + CD4 + regulatory T cells in immunological tolerance to self and non-self. Nat Immunol. 2005;6(4):345–352. 15785760 10.1038/ni1178

[41] Fan MY, Low JS, Tanimine N, et al Differential roles of IL-2 signaling in developing versus mature tregs. Cell Rep. 2018;25(5):1204–1213.e4. 30380412 10.1016/j.celrep.2018.10.002 PMC6289175

[42] Toomer KH, Lui JB, Altman NH, Ban Y, Chen X, Malek TR. Essential and non-overlapping IL-2Rα-dependent processes for thymic development and peripheral homeostasis of regulatory T cells. Nat Commun. 2019;10(1):1037. 30833563 10.1038/s41467-019-08960-1 PMC6399264

[43] Liu W, Putnam AL, Xu-yu Z, et al CD127 expression inversely correlates with FoxP3 and suppressive function of human CD4 + T reg cells. J Exp Med. 2006;203(7):1701–1711. 16818678 10.1084/jem.20060772 PMC2118339

[44] Ono M, Shimizu J, Miyachi Y, Sakaguchi S. Control of autoimmune myocarditis and multiorgan inflammation by glucocorticoid-induced TNF receptor family-related protein(high), Foxp3-expressing CD25 + and CD25- regulatory T cells. J Immunol. 2006;176(8):4748–4756. 16585568 10.4049/jimmunol.176.8.4748

[45] Bonelli M, Savitskaya A, Steiner CW, Rath E, Smolen JS, Scheinecker C. Phenotypic and functional analysis of CD4 + CD25- Foxp3 + T cells in patients with systemic lupus erythematosus. J Immunol. 2009;182(3):1689–1695. 19155519 10.4049/jimmunol.182.3.1689

[46] Coleman MM, Finlay CM, Moran B, Keane J, Dunne PJ, Mills KHG. The immunoregulatory role of CD4 ^+^ FoxP3 ^+^ CD25^−^ regulatory T cells in lungs of mice infected with Bordetella pertussis. FEMS Immunol Med Microbiol. 2012;64(3):413–424. 22211712 10.1111/j.1574-695X.2011.00927.x

[47] Prado C, de Paz B, López P, Gómez J, Rodríguez-Carrio J, Suárez A. Relationship between FOXP3 positive populations and cytokine production in systemic lupus erythematosus. Cytokine. 2013;61(1):90–96. 23022375 10.1016/j.cyto.2012.08.033

[48] Baek H, Ye M, Kang GH, et al Neuroprotective effects of CD4 + CD25 +Foxp3 + regulatory T cells in a 3xTg-AD Alzheimer’s disease model. Oncotarget. 2016;7(43):69347–69357. 27713140 10.18632/oncotarget.12469 PMC5342482

[49] Dansokho C, Ait Ahmed D, Aid S, et al Regulatory T cells delay disease progression in Alzheimer-like pathology. Brain. 2016;139(Pt 4):1237–1251. 26912648 10.1093/brain/awv408

[50] Faridar A, Vasquez M, Thome AD, et al Ex vivo expanded human regulatory T cells modify neuroinflammation in a preclinical model of Alzheimer’s disease. Acta Neuropathol Commun. 2022;10(1):144. 36180898 10.1186/s40478-022-01447-z PMC9524037

[51] Reynolds AD, Banerjee R, Liu J, Gendelman HE, Lee Mosley R. Neuroprotective activities of CD4 + CD25 + regulatory T cells in an animal model of Parkinson’s disease. J Leukoc Biol. 2007;82(5):1083–1094. 17675560 10.1189/jlb.0507296

[52] Beers DR, Henkel JS, Zhao W, et al Endogenous regulatory T lymphocytes ameliorate amyotrophic lateral sclerosis in mice and correlate with disease progression in patients with amyotrophic lateral sclerosis. Brain. 2011;134(Pt 5):1293–1314. 21596768 10.1093/brain/awr074 PMC3097891

[53] Oliveira LPG, Xavier RG, Nora CCV, et al Exhaustion profile on classical monocytes after LPS stimulation on Crohn’s disease patients. Hum Immunol. 2025;86(2):111257. 39952081 10.1016/j.humimm.2025.111257

[54] Byrne A, Reen DJ. Lipopolysaccharide induces rapid production of IL-10 by monocytes in the presence of apoptotic neutrophils. J Immunol. 2002;168(4):1968–1977. 11823533 10.4049/jimmunol.168.4.1968

[55] Han KH, Tangirala RK, Green SR, Quehenberger O. Chemokine receptor CCR2 expression and monocyte chemoattractant protein-1–mediated chemotaxis in human monocytes. Arterioscler Thromb Vasc Biol. 1998;18(12):1983–1991. 9848893 10.1161/01.atv.18.12.1983

[56] Romano M, Fanelli G, Tan N, et al Expanded regulatory T cells induce alternatively activated monocytes with a reduced capacity to expand T helper-17 cells. Front Immunol. 2018:9:1625. 30079063 10.3389/fimmu.2018.01625 PMC6062605

[57] Le Page A, Dupuis G, Frost EH, et al Role of the peripheral innate immune system in the development of Alzheimer’s disease. Exp Gerontol. 2018;107:59–66. 29275160 10.1016/j.exger.2017.12.019

[58] Leng F, Edison P. Neuroinflammation and microglial activation in Alzheimer disease: Where do we go from here? Nat Rev Neurol. 2021;17(3):157–172. 33318676 10.1038/s41582-020-00435-y

[59] Smith LK, He Y, Park JS, et al β2-microglobulin is a systemic pro-aging factor that impairs cognitive function and neurogenesis. Nat Med. 2015;21(8):932–937. 26147761 10.1038/nm.3898 PMC4529371

[60] Abellanas MA, Purnapatre M, Burgaletto C, Schwartz M. Monocyte-derived macrophages act as reinforcements when microglia fall short in Alzheimer’s disease. Nat Neurosci. 2025;28(3):1–10. 10.1038/s41593-024-01847-5 39762659

[61] Lok HC, Katzeff JS, Hodges JR, et al Elevated GRO-α and IL-18 in serum and brain implicate the NLRP3 inflammasome in frontotemporal dementia. Sci Rep. 2023;13(1):1–16. 37268663 10.1038/s41598-023-35945-4 PMC10238432

[62] Torres-Acosta N, O’Keefe JH, O’Keefe EL, Isaacson R, Small G. The rapeutic potential of TNF-α inhibition for Alzheimer’s disease prevention. J Alzheimers Dis. 2020;78(2):619–626. 33016914 10.3233/JAD-200711 PMC7739965

[63] Asken BM, Ljubenkov PA, Staffaroni AM, et al Plasma inflammation for predicting phenotypic conversion and clinical progression of autosomal dominant frontotemporal lobar degeneration. J Neurol Neurosurg Psychiatry. 2023;94(7):541–549. 36977552 10.1136/jnnp-2022-330866 PMC10313977

[64] Ou W, Yang J, Simanauskaite J, et al Biologic TNF-α inhibitors reduce microgliosis, neuronal loss, and tau phosphorylation in a transgenic mouse model of tauopathy. J Neuroinflammation. 2021;18(1):1–19. 34972522 10.1186/s12974-021-02332-7 PMC8719395

[65] Zhou YQ, Liu DQ, Chen SP, et al The role of CXCR3 in neurological diseases. Curr Neuropharmacol. 2019;17(2):142. 29119926 10.2174/1570159X15666171109161140 PMC6343204

[66] Jorfi M, Park J, Hall CK, et al Infiltrating CD8 + T cells exacerbate Alzheimer’s disease pathology in a 3D human neuroimmune axis model. Nat Neurosci. 2023;26(9):1489–1504. 37620442 10.1038/s41593-023-01415-3 PMC11184920

[67] Krauthausen M, Kummer MP, Zimmermann J, et al CXCR3 promotes plaque formation and behavioral deficits in an Alzheimer’s disease model. J Clin Invest. 2015;125(1):365–378. 25500888 10.1172/JCI66771 PMC4382235

[68] Gate D, Saligrama N, Leventhal O, et al Clonally expanded CD8 T cells patrol the cerebrospinal fluid in Alzheimer’s disease. Nature. 2020;577(7790):399–404. 31915375 10.1038/s41586-019-1895-7 PMC7445078

[69] Couto B, Forrest SL, Fearon C, et al Midbrain cytotoxic T cells as a distinct neuropathological feature of progressive supranuclear palsy. Brain. 2025;148(8):2650–2657. 40233181 10.1093/brain/awaf135 PMC12316005

