# Supplementary material for: Peripheral immune profiling in frontotemporal dementia
Source: Brain Commun. 2026 Mar 13;8(2):fcag089. doi: 10.1093/braincomms/fcag089 (PMC13037769; doi:10.1093/braincomms/fcag089)
Supplement: fcag089_Supplementary_Data [file fcag089_supplementary_data.docx]

Supplementary Material

**Supplementary Table 1.** **Demographic and clinical characteristics of the FTD cohort.**

Age (years), Sex (M = male, F = female), CDR (Clinical Dementia Rating), Age of onset (years), Disease duration (years), MMSE (Mini-Mental State Examination), PASS Rating Scale, and Education. N/A = not available.

| Diagnosis | Age | Sex | CDR | Age of onset | Disease duration | MMSE | PASS Rating Scale | Education |
| --- | --- | --- | --- | --- | --- | --- | --- | --- |
| bvFTD-1 | 63 | F | 3 | 59 | 4 | 2 | 2 | University |
| bvFTD-2 | 60 | F | 3 | 57 | 3 | 9 | 15.5 | University |
| bvFTD-3 | 60 | F | 3 | 55 | 5 | 11 | N/A | University |
| bvFTD-4 | 50 | M | 0.5 | 47 | 3 | 26 | 1 | Master’s |
| bvFTD-5 | 65 | F | 0.5 | 60 | 5 | 24 | 0 | University |
| bvFTD-6 | 73 | F | 1 | 66 | 7 | 28 | 1 | University |
| bvFTD-7 | 61 | F | 0.5 | 57 | 4 | 24 | 0.5 | High school |
| nfvPPA-1 | 66 | F | 1 | 57 | 9 | N/A | 10.5 | University |
| nfvPPA-2 | 79 | M | 0.5 | 71 | 8 | 22 | 8.5 | University |
| nfvPPA-3 | 67 | M | 0.5 | 61 | 6 | 9 | 11.5 | University |
| nfvPPA-4 | 74 | F | 1 | 70 | 4 | 16 | 8.5 | High school |
| nfvPPA-5 | 67 | M | 0.5 | 64 | 3 | 22 | 3.5 | University |
| nfvPPA-6 | 71 | F | 0.5 | 67 | 4 | 29 | 2 | Master’s |
| nfvPPA-7 | 81 | F | 0.5 | 76 | 5 | 27 | N/A | University |
| nfvPPA-8 | 51 | F | 0.5 | 46 | 5 | 15 | 15.5 | Postgraduate |
| nfvPPA-9 | 67 | F | 0.5 | 65 | 2 | 28 | 5 | University |
| nfvPPA-10 | 70 | F | 0.5 | 63 | 7 | 28 | 6 | High school |
| svPPA-1 | 60 | F | 1 | 58 | 2 | 18 | 4.5 | University |
| svPPA-2 | 67 | F | 0.5 | 64 | 3 | 26 | 6.5 | University |
| svPPA-3 | 69 | M | 1 | 66 | 3 | 26 | 5 | University |
| svPPA-4 | 69 | M | 3 | 66 | 3 | 4 | N/A | University |
| svPPA-5 | 57 | M | 2 | 55 | 2 | 20 | 8 | University |
| svPPA-6 | 64 | M | 0.5 | 53 | 9 | 18 | 9.5 | Master’s |
| svPPA-7 | 56 | F | 0.5 | 52 | 4 | 23 | 10 | University |
| svPPA-8 | 38 | M | 0.5 | 35 | 3 | 23 | 4.5 | High school |
| svPPA-9 | 59 | F | 1 | 52 | 7 | 0 | 17 | University |
| svPPA-10 | 67 | F | 0.5 | 65 | 2 | 24 | 5.5 | Master’s |

**Supplementary Table 2.** **Summary of participants’ distribution for each assay.**

HC (healthy control), FTD (Frontotemporal dementia), M = male, F = female. Group comparisons using unpaired t-test (Age, and CDR). Chi-square test of independence (and Fisher’s exact test) to calculate p value between HC and FTD groups based on sex distribution (male vs female).

| Assay | Diagnosis | n | Age (years)  Mean ± SD | Sex  M /F |
| --- | --- | --- | --- | --- |
| Treg suppressive function | **HC** | 12 | 66.17 ± 10.26 | 5 /7 |
|  | **FTD** | 21 | 65.81 ± 8.029 | 6 /15 |
|  | ***P value*** | | P = 0.9123 | P = 0.4713 |
| Tcell immunophenotype | **HC** | 14 | 64.07 ± 9.880 | 4 /10 |
|  | **FTD** | 17 | 64.53 ± 7.771 | 5 /12 |
|  | ***P value*** | | P = 0.8861 | P >0.999 |
| Monocyte transcriptomics | **HC** | 8 | 67.25 ± 8.924 | 3 /5 |
|  | **FTD** | 11 | 63.45 ± 11.27 | 4 /7 |
|  | ***P value*** | | P = 0.4417 | P >0.999 |
| Plasma proteomics | **HC** | 16 | 71.19 ± 8.304 | 8 /8 |
|  | **FTD** | 14 | 65.29 ± 7.384 | 7 /7 |
|  | ***P value*** | | P = 0.0505 | P >0.999 |

**Supplementary Table 3.** **List of the 37 inflammation-related genes significantly upregulated in FTD monocytes compared to HC.**

| **Gene** | **p_value** | **-log10(pvalue)** | **Mean of HC** | **Mean of FTD** | **Percent difference (%)** | **Direction** |
| --- | --- | --- | --- | --- | --- | --- |
| *BTG2* | 0.029711 | 1.527083 | 3.284 | 3.518 | 7.125457 | Up |
| *C1QA* | 0.000128 | 3.89279 | 2.244 | 2.647 | 17.959 | Up |
| *CCL2* | 0.033679 | 1.472641 | 1.691 | 1.888 | 11.64991 | Up |
| *CCL21* | 0.000202 | 3.694649 | 0.6949 | 1.053 | 51.53259 | Up |
| *CCL3L1* | 0.014954 | 1.825243 | 2.33 | 2.555 | 9.656652 | Up |
| *CD247* | 0.017559 | 1.7555 | 1.267 | 1.487 | 17.36385 | Up |
| *CD69* | 0.025625 | 1.591336 | 1.69 | 1.906 | 12.78107 | Up |
| *CD99* | 0.003943 | 2.404173 | 2.445 | 2.717 | 11.12474 | Up |
| *CLC* | 0.000017 | 4.769551 | 1.391 | 1.791 | 28.75629 | Up |
| *CTSL* | 0.014165 | 1.848783 | 2.16 | 2.387 | 10.50926 | Up |
| *CXCR3* | 0.000961 | 3.017277 | 1.751 | 2.08 | 18.78926 | Up |
| *CYP1B1* | 0.016961 | 1.770549 | 2.457 | 2.691 | 9.52381 | Up |
| *CYSLTR1* | 0.002672 | 2.573164 | 2.452 | 2.73 | 11.33768 | Up |
| *DES* | 0.012832 | 1.891706 | 1.825 | 2.069 | 13.36986 | Up |
| *ERCC3* | 0.034964 | 1.456379 | 1.871 | 2.078 | 11.0636 | Up |
| *ETS1* | 0.004149 | 2.382057 | 1.087 | 1.353 | 24.47102 | Up |
| *FCER2* | 0.048534 | 1.313954 | 1.827 | 2.009 | 9.961686 | Up |
| *GPR65* | 0.001183 | 2.927015 | 2.112 | 2.412 | 14.20455 | Up |
| *GUSB* | 0.047538 | 1.322959 | 2.873 | 3.057 | 6.404455 | Up |
| *GZMA* | 0.014597 | 1.835736 | 2.068 | 2.294 | 10.92843 | Up |
| *HDAC6* | 0.048791 | 1.31166 | 2.291 | 2.485 | 8.467918 | Up |
| *HDC* | 0.000657 | 3.182435 | 1.47 | 1.785 | 21.42857 | Up |
| *IL4I1* | <0.000001 | 6.029 | 0.9438 | 1.399 | 48.23056 | Up |
| *JUN* | 0.049565 | 1.304825 | 2.921 | 3.103 | 6.230743 | Up |
| *KLRK1* | 0.000755 | 3.122053 | 1.516 | 1.828 | 20.58047 | Up |
| *LTB* | 0.001197 | 2.921906 | 1.881 | 2.182 | 16.00213 | Up |
| *LTB4R* | 0.001658 | 2.780415 | 2.555 | 2.86 | 11.93738 | Up |
| *MS4A1* | 0.025928 | 1.586231 | 1.211 | 1.418 | 17.09331 | Up |
| *MS4A2* | 0.028987 | 1.537797 | 1.622 | 1.824 | 12.45376 | Up |
| *MX1* | 0.00129 | 2.88941 | 3.191 | 3.49 | 9.370103 | Up |
| *NCF2* | 0.047564 | 1.322722 | 3.685 | 3.872 | 5.074627 | Up |
| *NLRP3* | 0.026642 | 1.574433 | 2.495 | 2.705 | 8.416834 | Up |
| *PRG3* | 0.016377 | 1.785766 | 1.033 | 1.265 | 22.45886 | Up |
| *RHOC* | 0.006882 | 2.162285 | 2.975 | 3.241 | 8.941176 | Up |
| *SIGLEC1* | 0.000307 | 3.512862 | 2.293 | 2.627 | 14.56607 | Up |
| *VRK2* | 0.049677 | 1.303845 | 2.323 | 2.505 | 7.834697 | Up |
| *ZNF346* | 0.027836 | 1.555393 | 1.461 | 1.665 | 13.96304 | Up |

**Supplementary Table 4. List of the 40 inflammation-related genes significantly downregulated in FTD monocytes compared to HC.**

| **Gene** | **p_value** | **-log10(pvalue)** | **Mean of HC** | **Mean of FTD** | **Percent difference (%)** | **Direction** |
| --- | --- | --- | --- | --- | --- | --- |
| *ABCC8* | 0.049529 | 1.30514 | 0.3488 | 0.1667 | -52.2076 | Down |
| *ADAMTS2* | 0.019835 | 1.702568 | 2.022 | 1.806 | -10.6825 | Down |
| *ADAMTS9-AS2* | 0.020749 | 1.683003 | 1.586 | 1.367 | -13.8083 | Down |
| *ADCYAP1R1* | 0.034063 | 1.467717 | 1.357 | 1.161 | -14.4436 | Down |
| *ALOX15* | 0.016841 | 1.773632 | 1.485 | 1.259 | -15.2189 | Down |
| *CCL1* | 0.007019 | 2.153725 | 1.486 | 1.236 | -16.8237 | Down |
| *CCL11* | 0.000031 | 4.508638 | 0.7968 | 0.4103 | -48.5065 | Down |
| *CCL14* | 0.029295 | 1.533206 | 1.229 | 1.027 | -16.4361 | Down |
| *CCL25* | 0.000009 | 5.045757 | 1.464 | 1.053 | -28.0738 | Down |
| *CCL7* | 0.001684 | 2.773658 | 0.8163 | 0.5251 | -35.6732 | Down |
| *CD74* | 0.016694 | 1.77744 | 3.694 | 3.472 | -6.00975 | Down |
| *CDH1* | 0.02387 | 1.622148 | 0.3762 | 0.1667 | -55.6885 | Down |
| *CDH11* | 0.047537 | 1.322968 | 1.565 | 1.374 | -12.2045 | Down |
| *COL10A1* | <0.000001 | 10.3 | 0.7765 | 0.1667 | -78.5319 | Down |
| *COL14A1* | <0.000001 | 6.2 | 0.6288 | 0.1667 | -73.4892 | Down |
| *COL17A1* | 0.034313 | 1.464541 | 2.003 | 1.795 | -10.3844 | Down |
| *CTLA4* | 0.015201 | 1.818128 | 1.674 | 1.449 | -13.4409 | Down |
| *CXCL12* | 0.031517 | 1.501455 | 0.584 | 0.3846 | -34.1438 | Down |
| *ELL2* | <0.000001 | 12.67 | 2.108 | 1.427 | -32.3055 | Down |
| *EN1* | 0.010665 | 1.972039 | 1.585 | 1.349 | -14.8896 | Down |
| *FZD4* | 0.000088 | 4.055517 | 1.546 | 1.175 | -23.9974 | Down |
| *HLA-DPB1* | 0.018736 | 1.727323 | 4.002 | 3.785 | -5.42229 | Down |
| *HLA-DQA1* | 0.003475 | 2.459045 | 3.109 | 2.838 | -8.71663 | Down |
| *HLA-DQB1* | <0.000001 | 6.525 | 2.138 | 1.663 | -22.217 | Down |
| *HNF1B* | 0.00666 | 2.176526 | 1.583 | 1.332 | -15.856 | Down |
| *HOXD4* | 0.004043 | 2.393296 | 1.596 | 1.329 | -16.7293 | Down |
| *HSPG2* | 0.009521 | 2.021317 | 1.406 | 1.156 | -17.7809 | Down |
| *IL1A* | 0.033161 | 1.479372 | 1.515 | 1.318 | -13.0033 | Down |
| *KIAA0101* | 0.000043 | 4.366532 | 1.235 | 0.8556 | -30.7206 | Down |
| *KIF20A* | 0.012979 | 1.886759 | 1.758 | 1.527 | -13.1399 | Down |
| *NGF* | <0.000001 | 19.98 | 1.183 | 0.3163 | -73.2629 | Down |
| *NOS2* | 0.000278 | 3.555955 | 1.423 | 1.086 | -23.6824 | Down |
| *NOX1* | 0.00464 | 2.333482 | 0.9676 | 0.7051 | -27.129 | Down |
| *PDCD1* | 0.041492 | 1.382036 | 1.108 | 0.9192 | -17.0397 | Down |
| *PTPRB* | 0.026409 | 1.578248 | 1.601 | 1.387 | -13.3666 | Down |
| *PTPRK* | <0.000001 | 9.854 | 1.441 | 0.8095 | -43.8237 | Down |
| *RNASE3* | 0.017838 | 1.748654 | 2.251 | 2.031 | -9.77343 | Down |
| *SERINC2* | 0.025525 | 1.593034 | 2.456 | 2.249 | -8.42834 | Down |
| *TIMP3* | 0.002602 | 2.584693 | 1.855 | 1.575 | -15.0943 | Down |
| *VTCN1* | 0.037518 | 1.42576 | 1.688 | 1.495 | -11.4336 | Down |

**
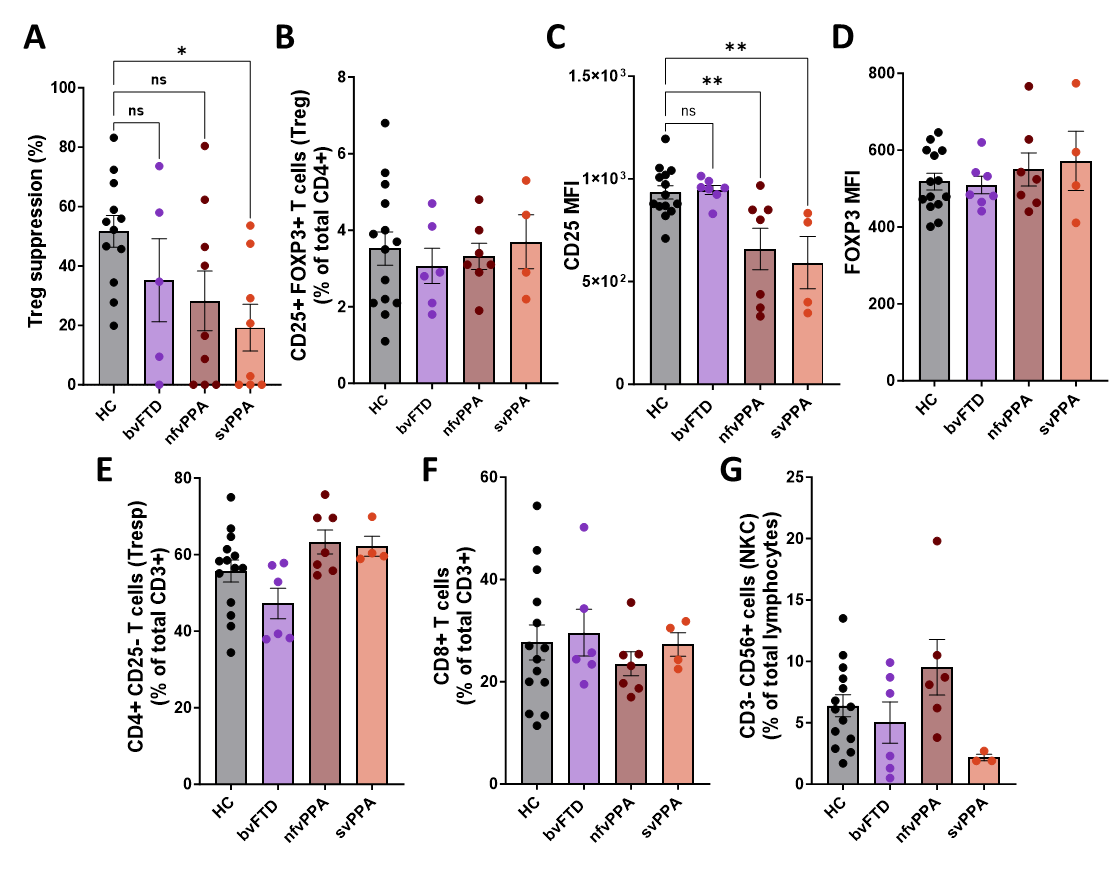
Supplementary Figure 1. Treg suppression analysis and Tcell phenotype in FTD subtypes vs HC. A)** Treg suppression is significantly reduced in the svPPA subtype when compared to HC individuals. **B)** The number of Tregs is comparable among the groups. **C)** CD25 MFI of Tregs is significantly reduced in nfvPPA and svPPA subtypes of FTD to that in HC and bvFTD. **D)** No significant changes were observed in FOXP3 MFI in Tregs. **E-G)** The number of Tresp, CD8T cells, and NKC were comparable among the three subtypes of FTD and HC. n = 12 HC, 5 bvFTD, 9 nfvPPA, and 8 svPPA (A); n = 14 HC, 6 bvFTD, 7 nfvPPA, and 4 svPPA (B-G). Graphs depicting mean ± SEM; One-way ANOVA, Dunnet’s multiple comparison test (A-D, F); nonparametric Kruskal-Wallis, Dunn’s multiple comparison test (E and G); *p<0.05; **p<0.01. Each individual data point represents a biological replicate.

**
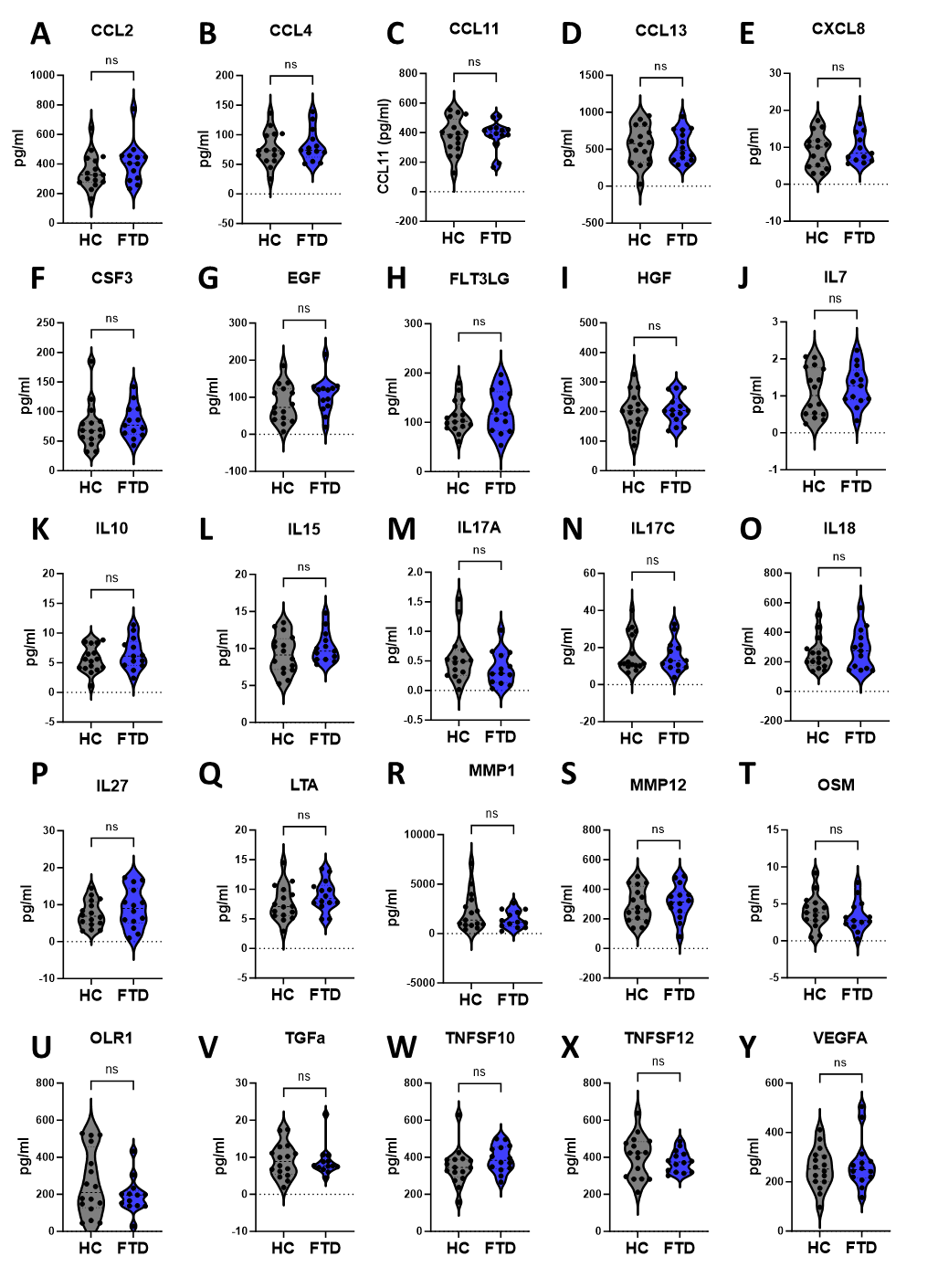
**

**Supplementary Figure 2. Levels of inflammatory markers in the plasma of FTD vs HC.** Levels of CCL2, CCL4, CCL11, CCL13, CXCL8, CSF3, EGF, FLT3LG, HGF, IL7, IL10, IL15, IL17A, IL17X, IL18, IL27, LTA, MMP1, MMP12, OSM, OLR1, TGFa, TNSF10, TNSF12, VEGFA **(A-Y)** showed no significant differences in plasma of FTD vs HC. Graphs depicting mean ± SEM; Unpaired Student t-test (B-E, G-I, K, L, N, P, Q, S, T, W, X) or nonparametric Mann-Whitney test (A, F, J, M, O, R, U, V, Y), ns = non-significant. Each individual data point represents a biological replicate n = 16 HC and 14 FTD individuals.

**
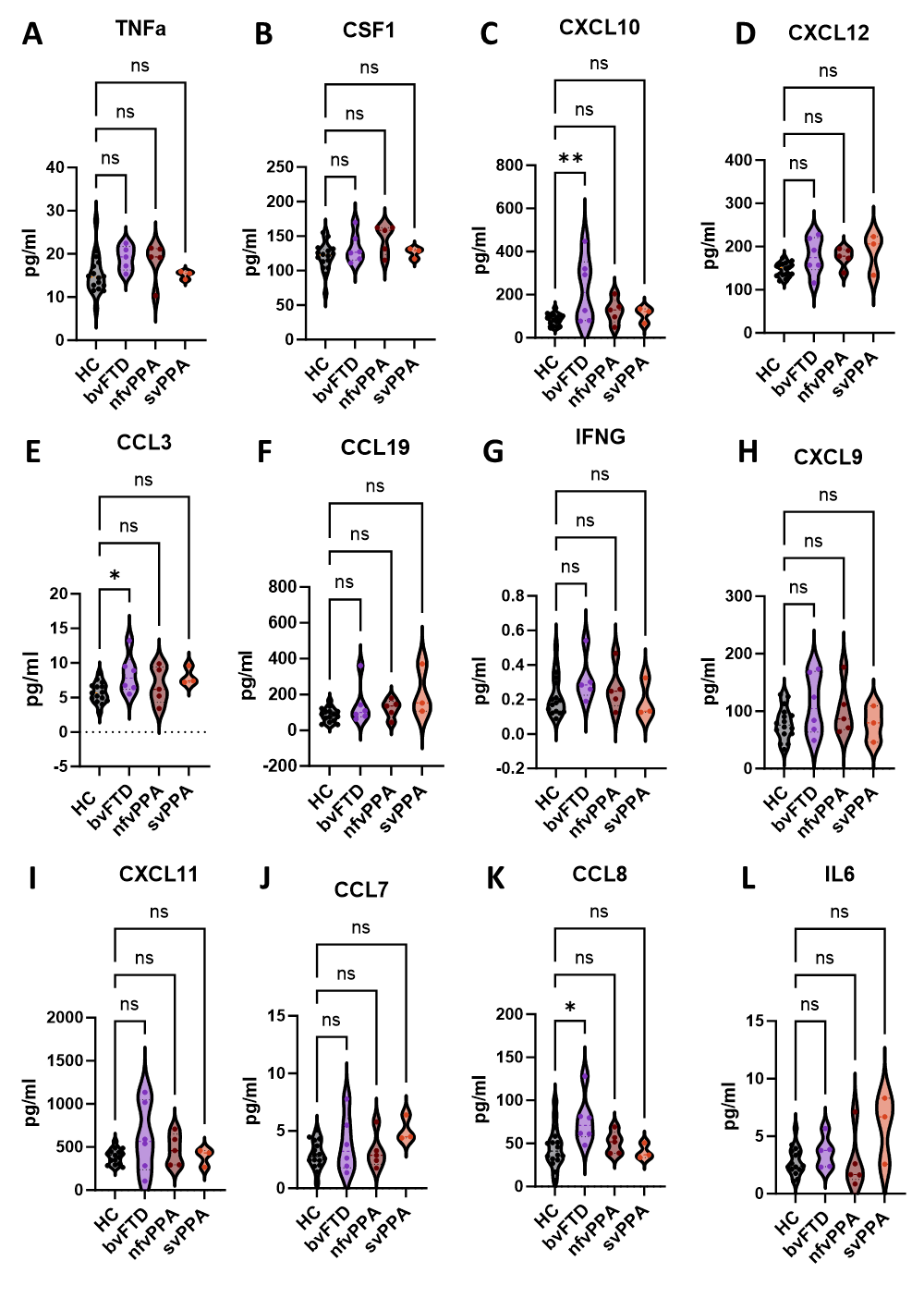
**

**
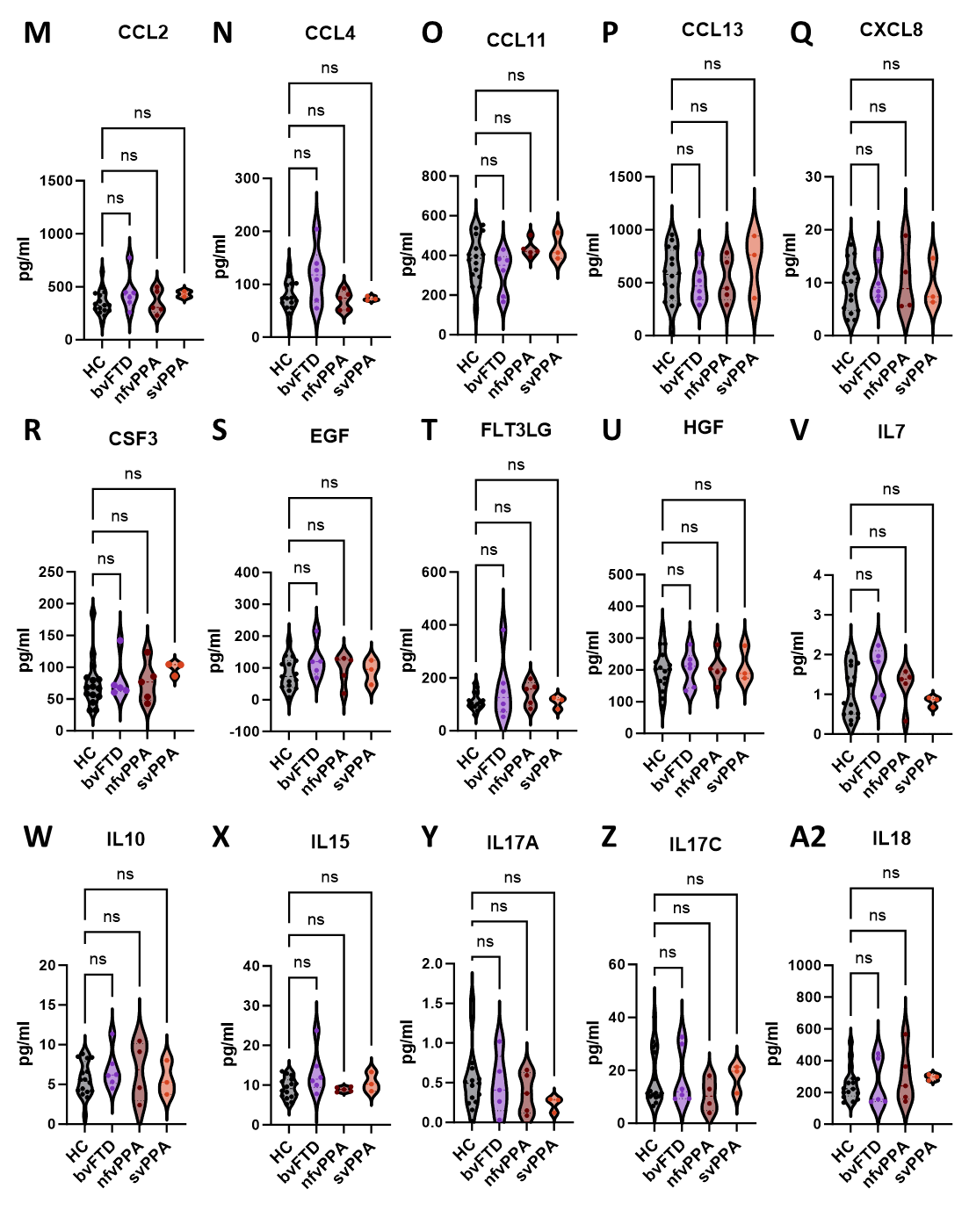
**

**
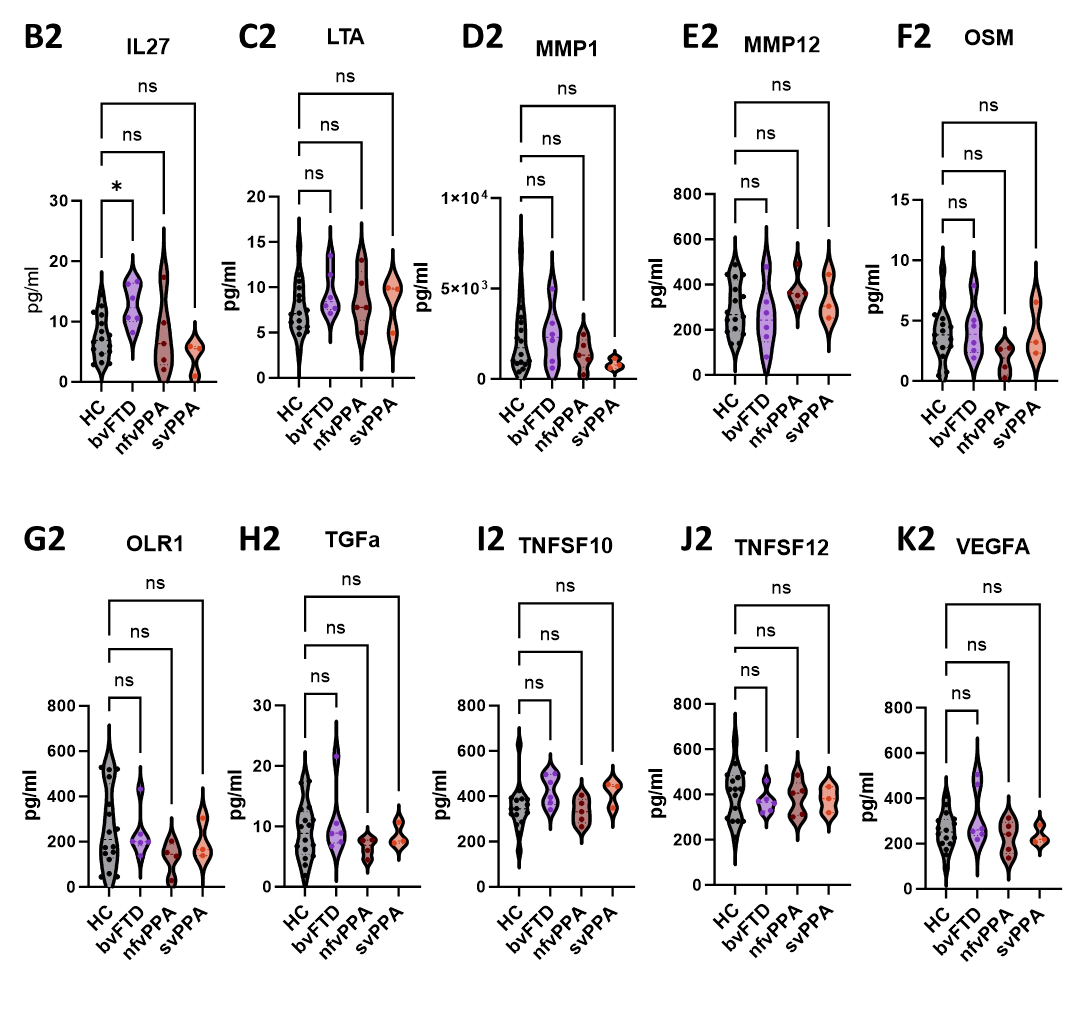
Supplementary Figure 3. Levels of inflammatory markers in the plasma of bvFTD, nfvPPA, and svPPA vs HC.** Levels of inflammatory cytokines and chemokines in plasma of FTD subtypes (bvFTD, nfvPPA, and svPPA) vs HC. Graphs depicting mean ± SEM; One-way ANOVA; Dunnet’s multiple comparison test (B-E, H-K, N-Q, S-U, W, X, Z, B2, E2, F2, I2, J2); nonparametric Kruskal-Wallis Dunn’s multiple comparison test (A, F, G, L, M, R, V, Y, A2, C2, D2, G2, H2, K2); *P<0.05; **P<0.01; ns = non-significant. Each individual data point represents a biological replicate; n = 16 HC, 6 bvFTD, 5 nfvPPA, and 3 svPPA individuals.
